# Long-Term Impact of Diffuse Traumatic Brain Injury on Neuroinflammation and Catecholaminergic Signaling: Potential Relevance for Parkinson’s Disease Risk

**DOI:** 10.3390/molecules29071470

**Published:** 2024-03-26

**Authors:** Ing Chee Wee, Alina Arulsamy, Frances Corrigan, Lyndsey Collins-Praino

**Affiliations:** 1 Cognition, Ageing and Neurodegenerative Disease Laboratory, School of Biomedicine, The University of Adelaide, Adelaide, SA 5005, Australia; ingchee.wee@adelaide.edu.au; 2 Neuropharmacology Research Laboratory, Jeffrey Cheah School of Medicine and Health Sciences, Monash University Malaysia, Bandar Sunway 47500, Selangor Darul Ehsan, Malaysia; alina.arulsamy@monash.edu; 3 Head Injury Lab, School of Biomedicine, The University of Adelaide, Adelaide, SA 5005, Australia; frances.corrigan@adelaide.edu.au

**Keywords:** traumatic brain injury, Parkinson’s disease, neuroinflammation, dopamine, noradrenaline

## Abstract

Traumatic brain injury (TBI) is associated with an increased risk of developing Parkinson’s disease (PD), though the exact mechanisms remain unclear. TBI triggers acute neuroinflammation and catecholamine dysfunction post-injury, both implicated in PD pathophysiology. The long-term impact on these pathways following TBI, however, remains uncertain. In this study, male Sprague-Dawley rats underwent sham surgery or Marmarou’s impact acceleration model to induce varying TBI severities: single mild TBI (mTBI), repetitive mild TBI (rmTBI), or moderate–severe TBI (msTBI). At 12 months post-injury, astrocyte reactivity (GFAP) and microglial levels (IBA1) were assessed in the striatum (STR), substantia nigra (SN), and prefrontal cortex (PFC) using immunohistochemistry. Key enzymes and receptors involved in catecholaminergic transmission were measured via Western blot within the same regions. Minimal changes in these markers were observed, regardless of initial injury severity. Following mTBI, elevated protein levels of dopamine D1 receptors (DRD1) were noted in the PFC, while msTBI resulted in increased alpha-2A adrenoceptors (ADRA2A) in the STR and decreased dopamine beta-hydroxylase (DβH) in the SN. Neuroinflammatory changes were subtle, with a reduced number of GFAP+ cells in the SN following msTBI. However, considering the potential for neurodegenerative outcomes to manifest decades after injury, longer post-injury intervals may be necessary to observe PD-relevant alterations within these systems.

## 1. Introduction

Traumatic brain injury (TBI) is a significant global cause of both mortality and disability [1]. In 2013, the United States alone recorded approximately 2.8 million TBI-related ED visits, hospitalizations, and deaths [2,3]. Worldwide, using the Global Burden of Disease Study 2016 data, 27.08 million (24.30–30.30 million) new cases of TBI were reported in 2016 compared to 2015 [4], although some estimates have suggested that this number may be as high as 50 million new cases globally each year [5]. While conventional belief once held that the effects of TBI could resolve over time, it is now well-recognized that TBI is, in fact, an ongoing disease process that may increase the risk for the later development of various neurodegenerative disorders, including Parkinson’s disease (PD), in a dose-dependent manner (for review, see Brett et al. 2022) [6]. In line with this, following a moderate–severe TBI, the likelihood of developing PD was higher compared to mild TBI (hazard ratio 1.50 vs. 1.24), with the risk of PD development increasing further with the cumulative effect of multiple TBIs in comparison to a single TBI (hazard ratio 1.87 vs. 1.45) [7].

Despite this, the precise neural mechanisms that underlie the relationship between TBI and the risk of PD development remain a subject of ongoing exploration. One such mechanism is the disruption in catecholamines, including dopamine (DA) and noradrenaline (NA), following injury. The loss of dopaminergic (DA) neurons within the substantia nigra (SN) is the pathological hallmark of PD [8,9,10,11,12]. This loss results in a disruption in the intricate balance of dopamine within the dorsal striatum (STR)—a region that receives DA projections directly from the SN [13,14,15]—subsequently leading to the manifestation of the cardinal motor symptoms observed in PD [8,16,17,18,19]. However, PD also involves the loss of noradrenergic (NA) neurons, with a 20–90% loss of NA neurons within the locus coeruleus (LC), a small brainstem structure that is the principal source of noradrenaline for the brain [20,21,22,23]. In fact, the LC is among the first brain regions to be affected in PD [24], with the loss of neurons in the LC beginning prior to, and to a greater extent, than that observed within the SN [20,24]. This depletion of LC neurons disrupts the supply of NA to regions such as the pre-frontal cortex (PFC), which is thought to contribute to various non-motor symptoms experienced by PD patients, including cognitive impairment, depression, and anxiety [8,25,26,27].

The depletion of dopamine and noradrenaline observed in PD can trigger a cascade of events involving the immune system, driving microglial and astrocyte activation and the concomitant release of pro-inflammatory molecules [28,29,30,31,32,33,34]. This neuroinflammatory process persists over time, in turn exacerbating the degeneration of DA and NA neurons and creating a self-perpetuating cycle of inflammation and neurodegeneration [35,36]. Evidence from a multitude of both in vitro and toxin-based animal model studies collectively underscores the vulnerability of SN-DA and LC-NA neurons in a chronic neuroinflammatory environment, with these neurons being among the most sensitive neurons in the brain to the effects of neuroinflammation [28,31,37,38]. There is also a growing body of neuropathological and biochemical evidence showing elevated levels of activated microglia and pro-inflammatory cytokines within the brains of individuals with PD, hinting at the pivotal involvement of neuroinflammation in disease pathology [39,40,41,42]. Further, the degeneration of LC-NA neurons leads to a loss of immune cell modulation by NA, further exacerbating SN-DA neuronal loss and disease pathogenesis (for review, see Butkovich et al. 2018) [43].

Critically, the disruption in catecholamines seen in PD is also present following TBI [44,45,46,47,48]. Studies in rodents have shown that, at 4 weeks following a moderate TBI induced via either controlled cortical impact or lateral fluid percussion, there are noticeable changes in dopamine metabolism, coupled with significant reductions in protein expression of the dopamine transporter (DAT), within both the STR and SN [48,49,50]. Similarly, disruptions in NA turnover rates were found in male Sprague-Dawley rats that sustained a moderate unilateral contusion induced by an air piston following the initial 30 min after injury [51]. Notably, a significant increase in NA turnover of 72% compared to uninjured controls was reported in the LC [51], with this upregulation found to decrease from 1 to 8 weeks post moderate diffuse injury in the same model [52]. Clinically, among 10 patients who experienced severe TBI several months prior, Single Photon Emission Computed Tomography (SPECT) imaging demonstrated significant disturbances in nigrostriatal function, as evidenced by reduced striatal DAT and D2-like receptor binding [53]. While there has been limited clinical research to date exploring changes in noradrenaline levels following TBI, the utilization of NA-targeting medications in TBI patients has shown promise, with improved recovery and reduced mortality [54,55,56]. Particularly noteworthy, a meta-analysis consisting of 17 randomized controlled trials investigating methylphenidate, a medication that enhances NA activity, suggested that methylphenidate administration led to improved processing speed in those with a history of TBI, particularly with prolonged drug duration, implying that NA level may be compromised following TBI, and that promoting NA activity could potentially restore this disrupted NA balance and lead to improvements in cognitive function [57].

Similarly, upregulation and prolonged neuroinflammation across different brain regions (including those relevant to PD pathology are some of the key consequential responses that the brain employs in response to the initial injury [58,59,60]. For instance, in mice with a diffuse axonal injury induced by the weight drop acceleration model, significant increases in proinflammatory cytokines, such as IL-1β, IL-6, and TNF-α, were observed in the medial PFC up to 9 days following a diffuse mild injury [61]. Furthermore, an increase in the transcription factor NF-κB and the proinflammatory enzymes Cox-2 and iNOS, as well as an upregulation in total microglial population, was demonstrated within the SN in mice at 4 weeks following a focal moderate TBI induced by controlled cortical impact [50]. Similarly, following a moderate midline fluid percussion injury, Sprague-Dawley rats demonstrated an elevation of proinflammatory cytokines, including IL-1β, IL-6, TNF-α, and CXCL1, in the cortex, STR, and SN, as early as 3–6 h post-injury [62]. This pro-inflammatory response appears to persist, with male Sprague-Dawley rats showing upregulated TSPO and CD45 gene expression in the SN, indicative of microglial activation, up to 28 days post-injury in a moderate diffuse injury induced by midline fluid percussion [48]. Similarly, within the STR, higher numbers of activated microglia have been reported up to 8 weeks following moderate injury induced by controlled cortical impact compared to sham uninjured animals [63]. Clinically, in a study of 10 patients with moderate–severe TBI, microglial activation has been shown to persist in several relevant brain regions, including the putamen, thalamus, and posterior limb of the internal capsule, even 17 years after the initial injury [64].

Taken together, this raises the possibility that persistent disruptions in catecholamine signaling and concomitant increases in neuroinflammation could, at least in part, help to set the stage for the later emergence of PD. However, despite this evident pathophysiological overlap, key questions remain. To date, only a limited number of preclinical studies have investigated the disruption of catecholamines following TBI, and the duration of observation often concludes prior to 6 months post-injury. In line with this, the longest follow-up investigated time point that we could identify in the literature was 28 weeks post-injury, where a 30% reduction in dopaminergic neurons was observed in animals subjected to moderate fluid percussion injury [65]. How catecholamine signaling may change at more chronic time points post-injury is not yet known. In contrast, there has been some long-term investigation of changes in neuroinflammation, with persistent microglial activity reported up to 24 months following TBI [59,66,67,68]. Noteworthy among these is the work of Mouzon and colleagues, demonstrating subtle, yet elevated, neuroinflammation in the corpus callosum from 12 to 24 months following both single and repetitive mild closed-head diffuse injuries [67,68]. Nevertheless, whether this post-TBI neuroinflammatory response is severity-dependent and how it may correlate with alterations in catecholamines remains to be explored.

As such, this study aimed to explore alterations in markers related to both catecholaminergic pathways and neuroinflammation within key PD-related regions (i.e., the STR, SN, and PFC at 12-months post-injury in a preclinical model of diffuse axonal injury of varying severities, including single mild, repetitive mild, and moderate–severe TBI. We hypothesized that there would be decreases in the integrity of both catecholaminergic pathways, with concomitant increases in markers of inflammation, at this chronic time point post-injury. This is significant because if markers of catecholaminergic signaling and neuroinflammation are chronically altered following TBI, monitoring such changes could potentially provide a means of identifying those at risk for later PD development, allowing for earlier identification, prior to the clinical onset of motor symptoms.

## 2. Results

### 2.1. Subtle Alterations in Neuroinflammation Observed following Moderate–Severe TBI at 12-Months Post-Injury

**Figure 1:**
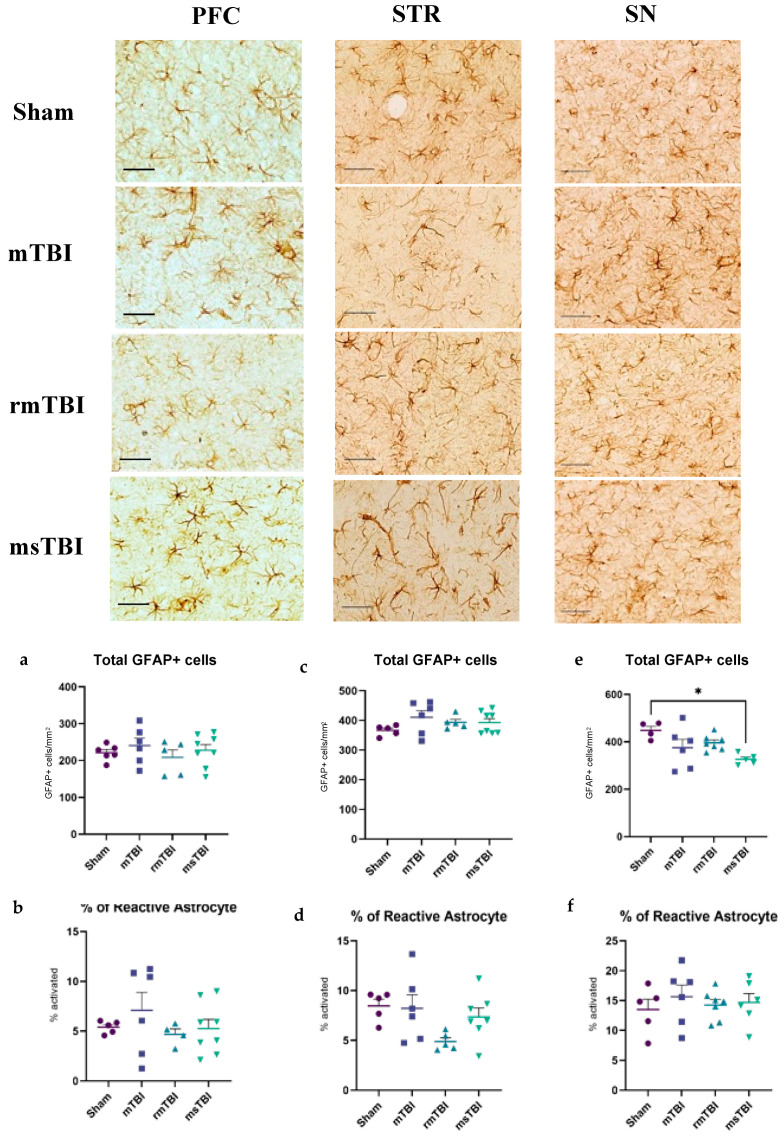
Neuroinflammation was assessed by glial fibrillary acidic protein (GFAP) at 12 months following different severities of injury. Representative images and the corresponding analysis of GFAP are shown in the left column for the prefrontal cortex (PFC), in the middle column for the striatum (STR), and in the right column for the substantia nigra (SN). (**a**,**b**) Total GFAP+ cells per mm^2^ and percentage of reactive astrocyte in PFC. (**c**,**d**) Total GFAP+ cells per mm^2^ and percentage of reactive astrocyte in STR. (**e**,**f**) Total GFAP+ cells per mm^2^ and percentage of reactive astrocyte in SN. Outliers were removed (*n* = 0–2 per group), and one-way ANOVA was performed. *n* = 4–8 per group. Data are presented as mean ± SEM, * *p* < 0.05. Scale bar = 50 μm.

Analysis of the total number of GFAP+ cells at 12 months post-injury found no significant difference in the PFC (F(3,21) = 0.57, *p* = 0.64) or the STR (F(3,20) = 1.292, *p* = 0.3) (Figure 1a,c), but an overall effect was found in the SN (F(3,18) = 4.293, *p* = 0.02), with the msTBI group showing a significantly lower number of GFAP+ cells/mm^2^ compared to the sham group (326.41 ± 22.17 cells/mm^2^ vs. 448.32 ± 35.27 cells/mm^2^, *p* = 0.013) (Figure 1e). No significant injury effect was found in any region for the percentage of reactive astrocytes (Figure 1b,d,f).

**Figure 2:**
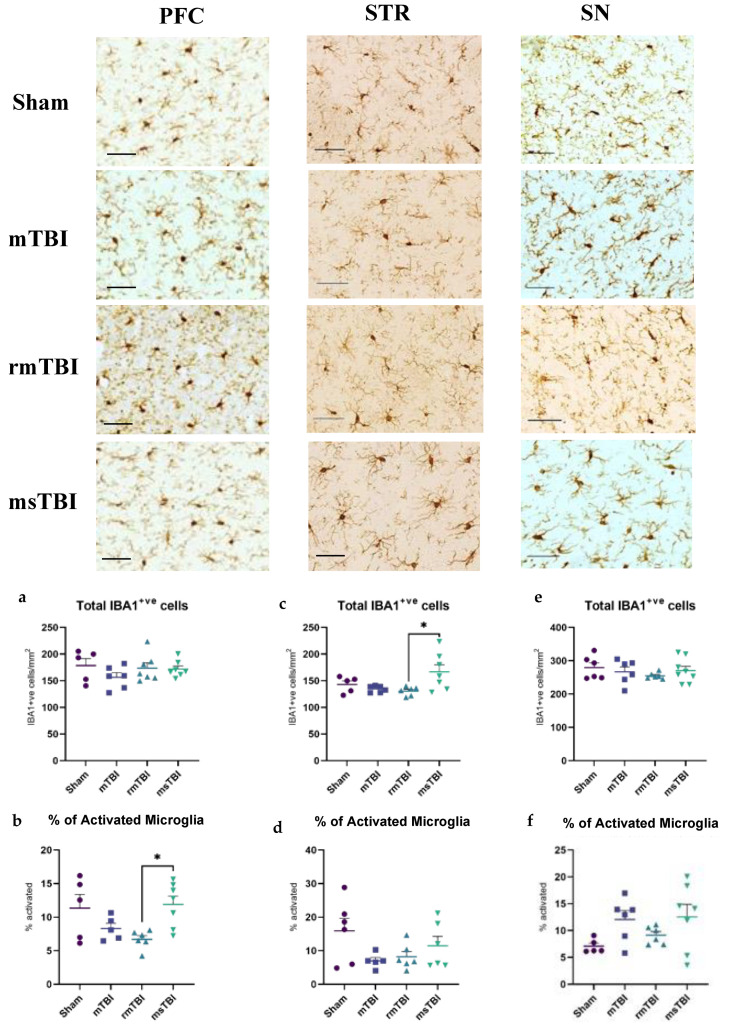
Neuroinflammation was assessed by ionized calcium-binding adaptor molecule 1 (Iba1) at 12 months following different severities of injury. Representative images and the corresponding analysis of IBA1 are shown in the left column for the prefrontal cortex (PFC), in the middle column for the striatum (STR), and in the right column for the substantia nigra (SN). (**a**,**b**) Total IBA1+ cells per mm^2^ and percentage of activated microglia in PFC. (**c**,**d**) Total IBA1+ cells per mm^2^ and percentage of activated microglia in STR. (**e**,**f**) Total IBA1+ cells per mm^2^ and percentage of activated microglia in SN. Outliers were removed (*n* = 0–1 per group) and one-way ANOVA was performed. *n* = 5–8 per group. Data are presented as mean ± SEM, * *p* < 0.05. Scale bar = 50 μm.

**Figure 3:**
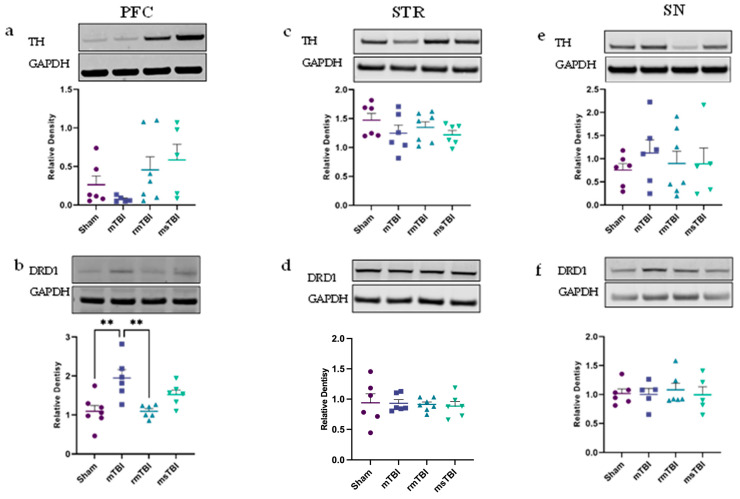
Dopaminergic pathway was assessed by TH and DRD1 at 12 months post different severities of injury within the PFC (**Left**), STR (**Middle**) and SN (**Right**). (**a**,**c**,**e**) Relative density of TH. (**b**,**d**,**f**) Relative density of Drd1. GAPDH was used as a housekeeper protein for analysis. Outliers were removed (*n* = 0–2 per group), and one-way ANOVA was performed. Data are presented as mean ± SEM, ** *p* < 0.01. *n* = 5–7 per group. Representative images of the Western blots were extracted from Image Studio Lite version 5.2.

For microglia, no significant difference in the number of IBA1+ cells was noted in the PFC (F(3,21) = 0.99, *p* = 0.42) or the SN (F(3,22) = 0.61, *p* = 0.62) at 12 months post-injury (Figure 2a,e). Notably, a significant effect of injury was identified in the STR (F(3,20) = 0.41, *p* = 0.02) (Figure 3c). Sham, mTBI, and rmTBI animals had similar numbers of IBA1+ cells (142.9 ± 15.13 cells/mm^2^, 134.61 ± 6.17 cells/mm^2^, 130.5 ± 8.37 cells/mm^2^, respectively), while an increase was observed in msTBI animals (166.8 ± 34.6 IBA1+ cells/mm^2^). However, post hoc analysis indicated that this elevation was not significantly different from shams (*p* > 0.05; Figure 2c).

No significant differences in the % activated microglia were noted in the STR (F(3,19) = 2.32, *p* = 0.11) or the SN (F(3,20) = 2.29, *p* = 0.11) at this time point following injury (Figure 2d,f); however, a significant effect was found in the PFC (F(3,19) = 4.11, *p* = 0.02) (Figure 2b). Sham and msTBI animals had a similar number of % activated microglia (11.34 ± 0.46% and 11.88 ± 3.30%), with lower numbers in the mTBI (8.30 ± 1.75%) and rmTBI animals (6.70 ± 1.38%). Post hoc analysis, however, found no significant differences relative to shams (*p* > 0.05; Figure 2b). A summary of the changes observed for all inflammatory markers is presented in Table 1.

### 2.2. DRD1 Elevation Observed in PFC, but Not STR or SN, following Single Mild TBI at 12 Months Post-Injury

To assess potential alterations in the dopaminergic pathway at 12 months following TBI, we conducted Western blot analysis of relevant proteins involved in this pathway. These included TH (tyrosine hydroxylase), responsible for dopamine production [69,70], and one of the dopamine D1-like receptors, DRD1. No alteration in the relative expression of TH was found in any of the regions examined, including PFC (F(3,19) = 2.01, *p* = 0.15), STR (F(3,21) = 0.08, *p* = 0.97), and SN (F(3,20) = 0.34, *p* = 0.8) (Figure 3a,c,e, Supplementary Figure S2). However, a significant change was observed for the relative expression of DRD1 in the PFC only (F(3,21) = 7.56, *p* < 0.01) (Figure 3b), primarily driven by an increase in mTBI animals (1.95 ± 0.53), such that they were significantly different from shams (1.09 ± 0.39, *p* = 0.002) and rmTBI animals (1.09 ± 0.17, *p* = 0.003), but not msTBI rats (1.52 ± 0.29, *p* = 0.22) (Figure 3b, Table 2, Supplementary Figure S3). To note, other players in the dopaminergic pathway, such as another member of the D1-like receptor family, DRD5, and D2-like receptors, including DRD2 and DRD4, as well as the dopamine transporter (DAT), were included in the original experimental plan. However, due to the poor antibody quality, further analysis of their expression levels and functional roles could not be reliably conducted for the purposes of this study.

### 2.3. Moderate–Severe TBI Leads to Chronic Changes in STR ADRA2A and SN DβH Levels at 12 Months Post-Injury

**Figure 4:**
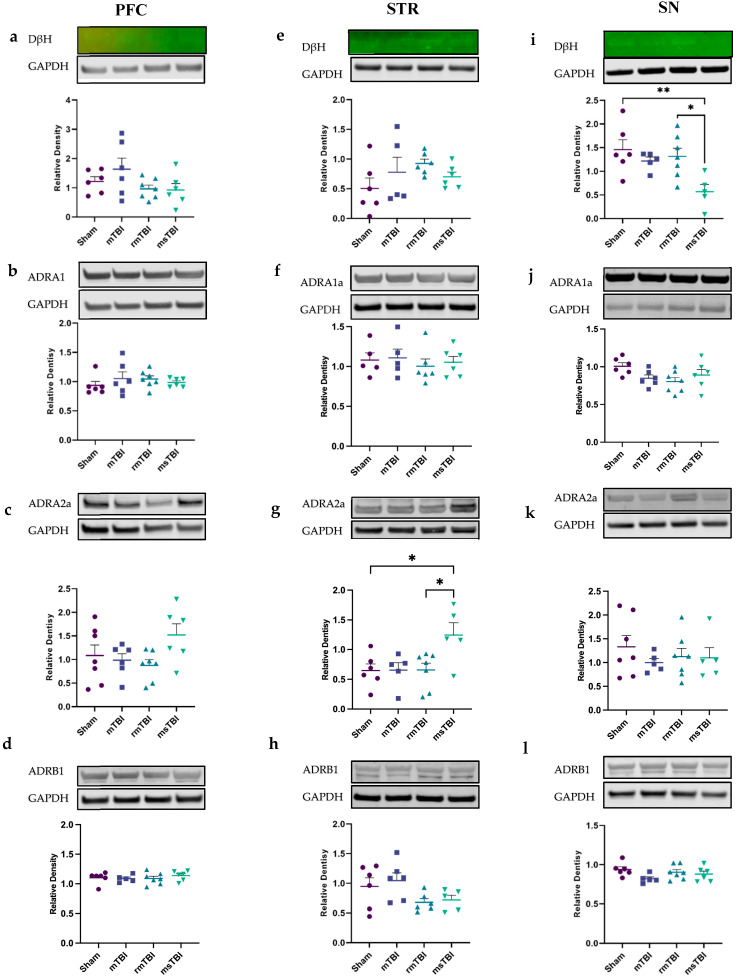
Noradrenergic pathway was assessed by DβH, ADRA1a, ADRA2a, and ADRB1 at 12 months post different severities of injury within PFC (left), STR (middle) and SN (right). (**a**,**e**,**i**) Relative density of DβH. (**b**,**f**,**j**) Relative density of ADRA1a. (**c**,**g**,**k**) Relative density of ADRA2a. (**d**,**h**,**l**) Relative density of ADRB1. GAPDH was used as a housekeeper protein for analysis. Outliers were removed (*n* = 0–2 per group) and one-way ANOVA was performed. Data are presented as mean ± SEM, * *p* < 0.05, ** *p* < 0.01. *n* = 5–7 per group. Representative images of the Western blots were extracted from Image Studio Lite. For clearer DBH images, please refer to Supplementary Figure S4.

The analysis of markers within the noradrenergic pathway included the examination of DβH (dopamine beta-hydroxylase), an enzyme responsible for noradrenaline production [71], as well as the noradrenergic receptors ADRA1a, ADRA2a, and ADRB1. Evaluation of noradrenaline synthesis using DβH showed no significant differences in the PFC (F(3,21) = 1.89, *p* = 0.16) (Figure 4a) or the STR (F(3,19) = 1.35, *p* = 0.29) (Figure 4e). However, an injury effect was observed in the SN (F(3,19) = 4.95, *p* = 0.01) (Figure 4i). Further post hoc analysis revealed that the msTBI group exhibited significantly lower relative expression of DβH (0.57 ± 0.39), compared to both the sham (1.46 ± 0.51, *p* = 0.009) and rmTBI (1.32 ± 0.45, *p* = 0.026) groups, while no significant difference was observed compared to the mTBI group (1.22 ± 0.19, *p* = 0.089). As the DβH bands are not visibly apparent on the grey-scale blot (Supplementary Figure S4), their position and intensity were cross-verified through the fluorescent intensity analysis tab in the Licor image studio and are presented here (Figure 4a,e,i).

Conversely, no changes were detected in ADRA1a levels in the PFC (F(3,21) = 0.59, *p* = 0.63), STR (F(3,18) = 1.07, *p* = 0.39) or SN (F(3,21) = 2.44, *p* = 0.09) (Figure 4b,f,j; Supplementary Figure S5). Similarly, for ADRB1, no changes were noted within any of these regions (PFC: F(3,20) = 0.42, *p* = 0.74; STR: F(3,19) = 2.48, *p* = 0.09; F(3,20) = 1.88, *p* = 0.17) (Figure 4d,h,l; Supplementary Figure S7). While there did appear to be a trend towards reduction in this receptor in the STR following both rmTBI and msTBI, this failed to reach statistical significance. However, a significant effect of injury was observed in ADRA2a, specifically in the STR region (F(3,19) = 4.07, *p* = 0.02) (Figure 4g), with msTBI animals expressing significantly higher levels of ADRA2a (1.24 ± 0.47) compared to the sham (0.65 ± 0.28, *p* = 0.04) or rmTBI (0.66 ± 0.31, *p* = 0.03) groups. In contrast, no such effect was observed in either the PFC (F(3,18) = 2.21, *p* = 0.13) or SN (F(3,20) = 0.52, *p* = 0.68) (Figure 4c,k) (Supplementary Figure S6).

### 2.4. Expression of COMT Was Not Altered by Chronic TBI

**Figure 5:**
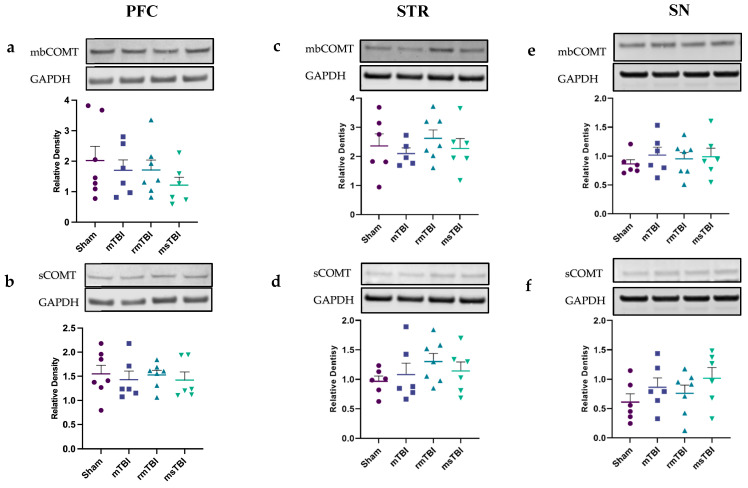
Catecholamine degradation rate was assessed by COMT at 12 months post different severities of injury within PFC (**left**), STR (**middle**) and SN (**right**). (**a**,**c**,**e**) Relative density of mbCOMT. (**b**,**d**,**f**) Relative density of sCOMT. GAPDH was used as a housekeeper protein for analysis. Data are presented as mean ± SEM. Outliers were removed (*n* = 0–2) and one-way ANOVA was performed. *n* = 5–7 per group. Representative images of the Western blots were extracted from Image Studio Lite.

At 12 months post-injury, there were no changes in COMT, the enzyme that degrades dopamine and noradrenaline [72], in any region, regardless of whether soluble (sCOMT) or membrane-bound (mbCOMT) was examined in the PFC (sCOMT: F(3,22) = 0.17, *p* = 0.92; mbCOMT F(3,22) = 0.81, *p* = 0.5); STR (sCOMT F(3,21) = 0.9323, *p* = 0.44; mbCOMT: F(3,20) = 0.45, *p* = 0.72); and SN (sCOMT F(3,21) = 1.15, *p* = 0.35; mbCOMT F(3,21) = 0.3, *p* = 0.83) (Figure 5; Supplementary Figure S8). A summary of the changes observed for all catecholaminergic markers is presented in Table 2.

## 3. Materials and Methods

### 3.1. Animals

This study utilized previously generated tissue from fifty-four adult male Sprague-Dawley rats (10–12 weeks, 420–480 g), originally obtained from Laboratory Animal Services (The University of Adelaide, AU). Animals were housed under conventional laboratory conditions (2 animals per cage), with a 12 h light–dark cycle and access to food and water ad libitum. All animals were housed in standard wireframe open-top cages on corncob bedding with a variety of enrichment provided within the cage, including wooden blocks, toilet paper roll tubes, and empty boxes. This study was performed under the approval of the University of Adelaide Animal Ethics Committee (M-2015-187).

### 3.2. Experimental Groups and Study Design

**Figure 6:**
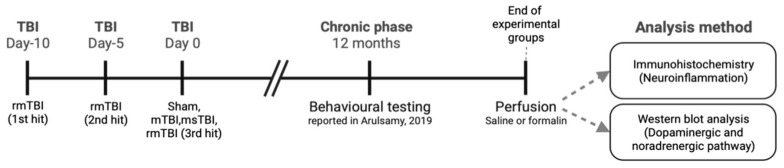
Experimental design of the study. During the chronic phase (12 months post-injury), animals underwent neurological and behavioral testing [73]. At the end of behavioral testing, the animals underwent perfusion using either saline or formalin fixative. This perfusion process was carried out for subsequent analyses: Western blot and immunohistochemistry, respectively.

Animals were randomly allocated to receive either sham surgery (*n* = 14), a single mild TBI (smTBI) (*n* = 12), repetitive mild TBI (rmTBI) (*n* = 14), or moderate–severe TBI (msTBI) (*n* = 14). To control for anesthesia and analgesia exposure, animals in the sham group received identical procedures excluding injury, with repetitive sham animals receiving the incision three times, with 5-day intervals between each incision. At 12 months following the last TBI or sham procedure, each animal underwent a comprehensive functional battery assessing motor, neuropsychiatric, and cognitive function as previously reported [73,74]. This 12-month time point was selected for investigation as no study to date has investigated changes in catecholaminergic signaling beyond 28 weeks post-injury. Further, this represents a life stage comparable to early middle age in humans [75], which is an important period for investigating underlying pathophysiological alterations that may be indicative of later risk of PD development. This is particularly relevant given that pathological changes can occur in the brain for decades prior to the onset of the motor symptoms of PD [76]. At the end of the behavioral assessment, animals were randomly assigned for either (1) molecular analysis or (2) immunohistochemistry using simple randomization procedures (computerized random numbers) (Figure 6).

### 3.3. Injury Protocol and Postoperative Care

Briefly, animals were injured with the Marmarou impact-acceleration injury method [77], an extensively validated diffuse injury model [78]. The msTBI group was intubated and mechanically ventilated, while all other groups were maintained on anesthesia via a nose cone. A 450 g weight was released from either 2 m (msTBI) or 0.75 m (mTBI, rmTBI) onto a metal disc affixed centrally to the rat’s skull, with the msTBI animals exposed to 10 min of hypoxic conditions (2 L/min nitrogen; 0.2 L/min oxygen) to imitate the clinical effects of more severe head trauma [73,74,78]. The rmTBI animals received 3 hits in 10 days (5-day intervals between each injury). After the TBI surgery, animals underwent regular weight checks and multiple daily welfare assessments for up to 2 weeks post-injury to monitor animal health. As confirmation of the success of the injury, animals subjected to msTBI displayed balance and motor coordination deficits on the rotarod test compared to sham animals, persisting up to 4 days post-injury, with no significant difference for the subsequent 3 months (refer to Arulsamy et al. 2018 for further details) [79]. We maintained ongoing attention to the well-being of the animals throughout the 12-month study duration. Unfortunately, four animals were lost during this time due to age-related health complications. Additional details can be found in Arulsamy et al. [73]. Previous studies have reported on impairments in cognitive flexibility following msTBI [73], as well as decreases in locomotion (following both rmTBI and msTBI) and anxiety (following mTBI [80]), when compared to age-matched sham animals within this cohort.

### 3.4. Immunohistochemistry

A subset of animals (6 sham, 6 mTBI, 7 rmTBI, and 8 msTBI) were transcardially perfused with formalin, and the brain was removed. Brains were exposed to 30% sucrose solution for cryoprotection and then segmented into 2–3 mm blocks. Blocks were embedded in Optimal Cutting Temperature compound (Tissue-Tek O.C.T. compound, Proscitech, Kirwan, Australia) and snap-frozen using freezing isopentane (2-methylbutane, Sigma Aldrich, St. Louis, MO, USA, M32631). Coronal sections of 20 μm were obtained from the desired locations for each animal using a cryostat and a rat brain atlas [81] (Table 3). The frozen sections were mounted on slides and stored in a −20 °C freezer.

For immunohistochemistry, slides were air-dried overnight at room temperature and rehydrated using ethanol. Endogenous peroxidases were blocked with 0.5% hydrogen peroxide in methanol (Thermo Fisher, Waltham, MA, USA), and antigen retrieval was performed using citrate buffer. Two phosphate-buffered saline (PBS) washes of 3 min each were performed, followed by a 30 min incubation in normal horse serum (1:30, Thermo Gibco, Waltham, MA, USA) and an overnight incubation with the primary antibody (Table 4).

On the following day, the slides were washed with 0.1% triton-X-100 in PBS, followed by the application of the appropriate biotinylated secondary antibody (1:250, Vector Laboratories, Newark, California, United States) for 30 min. Three PBS washes of 3 min each were performed between the application of the secondary and tertiary antibodies. Subsequently, the tertiary antibody streptavidin peroxidase conjugate (1:1000, Vector) was applied and incubated for one hour, and the bound antibody was detected using 3,3′-Diaminobenzidinetetrahydrochloride (1:50, Sigma Aldrich, St. Louis, MO, USA) for 7 min. After staining, the sections were mounted with coverslips using DPX mountant (Sigma Aldrich). The slides were air-dried in a fume hood for at least 2 days before being scanned with a Nanozoomer (Hamamatsu, Shizuoka, Japan) at 7 layers, with a separation of 1 μm between layers. The scanned images were viewed using the associated NDP view software (version 2).

### 3.5. Image Analysis

For analysis, 3 × 20× images were taken from the clearest layer in the region of interest (Table 1) and were exported and stacked with Image J to allow visualization of cell structure. GFAP immunoreactivity was assessed quantitatively by counting the reactive and immuno-positive cells per mm^2^ within the same area. For IBA1 analysis, all the stacked images were exported and analyzed by using the HALO image analysis platform (Indica Labs, Albuquerque, NM, USA). Analysis settings were based on the microglial activation module (v1.2) for automated counting. In brief, the images were set according to the scanning resolution, 20× mode, 0.46μm/pixel. After that, the total microglia population and activation state were determined based on morphological parameters of IBA1+ cells (Table 4, Supplementary Figure S1). The experimenter was blinded to the experimental group during the analysis.

### 3.6. Western Blot

Another subset of animals (8 sham, 6 mTBI, 7 rmTBI, and 6 msTBI) were transcardially perfused with 9% cold saline. The brains were dissected and immediately frozen in liquid nitrogen and stored at −80 °C. For further investigation, a 2–3 mm region of interest was cut from each brain tissue sample (Table 1). These samples were sonicated in freshly prepared RIPA buffer (20 mM Tris-HCl pH 7.5, 2 mM EDTA, 0.5 mM EGTA, 140 mM 2-mercaptoethanol) supplemented with a protease inhibitor (cOmplete Mini, EDTA free, Sigma, St. Louis, MO, USA). Each sample underwent three bursts of a 10 s duration (at least 1 min gap) using a sonicator probe. After sonication, the homogenized samples were centrifuged at 14,000 rpm and 4 °C for 30 min, and the supernatant was collected. The protein concentration of each sample was determined using the Pierce BCA Protein Assay (Thermo Scientific, Waltham, MA, USA) by measuring the absorbance at 650 nm.

For Western blot analysis, samples were prepared by adding sample buffer (Bolt^TM^, Tallinn, Estonia, 4× LDS Sample buffer) and reducing agent (Bolt^TM^, 10× sample reducing agent) to achieve a concentration of 1 mg/μL. A total of 20 mg of protein was loaded in each well. To maintain consistency across different blots, a sham sample was utilized as a standard, resulting in the inclusion of the sham number in the analysis (*n* = 7). Gel electrophoresis was performed using Bolt 4–12% Bis–Tris Plus gels (Invitrogen, Carlsbad, CA, USA) to separate the protein samples, followed by transfer onto a PVDF membrane using the iBlot 2 Dry Blotting System (Invitrogen). The membranes were then incubated with 5% milk-Tris buffer saline with 0.1% Tween 20 for 2 h and then with the appropriate primary antibody (Table 5) diluted in 2% bovine serum albumin (BSA, Sigma Aldrich) overnight at 4 °C. Afterward, the membranes were incubated with the corresponding secondary antibodies (donkey anti-rabbit, 1:10,000 and donkey anti-chicken, 1:10,000) for 2 h at room temperature. The western blots were imaged using an Odyssey Infrared Imaging System (model 9120; software version 3.0.21) (LI-COR, Inc., Lincoln, NE, USA) at a resolution of 169 μm. Quantitative analysis of the band signals was performed using Image Studio Lite version 5.2.

### 3.7. Statistics

Data analysis was performed using Prism software (GraphPad v.9.0). Statistical outliers were identified and removed based on the interquartile range in a box plot in SPSS. An ordinary one-way ANOVA (Analysis of Variance) with Tukey’s multiple comparison post hoc test was conducted to determine statistical significance. All values are presented as mean ± SEM, and a significance level of *p* < 0.05 was considered statistically significant.

## 4. Discussion

Alterations in catecholamines, along with the persistent upregulation of neuroinflammatory processes, are acknowledged to play pivotal roles in the pathophysiology of PD, and are equally implicated in the aftermath of TBI [82]. TBI is a known risk factor for the later development of PD, with risk varying based on initial injury severity [7,83,84]. To our knowledge, this was the first study to assess whether severity-dependent alterations in markers of DA and NA signaling and microglial and astrocytic reactivity within key regions implicated in PD, the PFC, STR, and SN persist up to 12 months after injury in an experimental model of diffuse axonal injury. This is significant, as such changes could potentially set the stage for the progression from TBI to PD.

Overall, the findings of this study indicate that, across the multiple DA and NA markers examined, following msTBI, only the NA pathway was disrupted, with a decrease in DβH within the SN and an increase in the NA receptor ADR2A within the STR, an effect that was not noted in animals with a milder initial injury. In comparison, only mTBI animals showed alterations in dopaminergic signaling, and this was only associated with an increase in DRD1 in the PFC, an effect not seen in any other injury group. No other changes in any dopaminergic or noradrenergic marker were noted across the three brain regions examined following injury, although it is important to note that we were not able to assess D2-like family receptors in this study. Similarly, in the evaluation of the glial response, the only significant difference relative to shams was a decrease in astrocytes, as detected by GFAP, in msTBI animals within the SN only. Taken together, the results of the current study seem to suggest that, at 12 months following the initial insult, diffuse axonal injury has minimal effect on catecholamines and the neuroimmune response, with subtle differences seen in mild compared to moderate–severe TBI.

The most notable finding in this study was that moderate–severe TBI led to an increase in ADRA2A within the STR and a decrease in DβH in SN at 12 months post-injury, while no such effects were seen in the PFC or any aspect of the DA pathway at this injury severity. DβH is an enzyme crucial for synthesizing NA [85,86], with noradrenergic neurons known to project from the LC to the SN [87,88,89,90], raising disruption of the LC as one potential explanation for this alteration. The LC is known to be affected by TBI, with an acute increase in NA turnover in both focal and diffuse models of injury [51,52], followed by a decrease more chronically (i.e., up to 8 weeks post-injury) in the Marmarou weight drop model [52]. Consistent disruption in NA levels is also evident from clinical work; although not LC specific, plasma and cerebrospinal fluid (CSF) samples collected from severe TBI patients showed significantly increased NA levels from 1 to 14 days following injury [47], although how NA levels may change more long-term following injury has not yet been investigated. While no changes were noted in either ADRA1a or ADRB1 receptors in the current study, it may be that WB is not the most effective tool for investigating such changes. In line with this, quantitative flow cytometry (qFlow) has recently emerged as one of the best techniques for probing changes in the abundance of plasma membrane receptors (for a detailed protocol, see recent work by Fang and colleagues (2022)) [91], and may, therefore, be a useful tool for investigating alterations in these receptors in future work. This may be particularly relevant for probing potential long-term alterations in ADRB1 receptors further, which appeared to decline in the STR following both rmTBI and msTBI, although this failed to reach statistical significance using the methods employed in the current study and should, therefore, be interpreted with caution.

It is noteworthy that prior research from our own laboratory has identified mild cognitive deficits at 12 months post-moderate–severe TBI within the same animal cohort [73], suggesting a potential link between the observed alteration in NA levels following TBI and these cognitive impairments. In fact, NA is a neurotransmitter that plays a fundamental role in response to stress, mood regulation, attention, and cognitive functions, including executive function, cognitive flexibility, learning, and memory [92]. Interestingly, prior research on Alpha-2A adrenergic agonists (which activate ADRA2A receptors and inhibit NA release) has consistently demonstrated their potential to enhance spatial working memory performance and cognitive flexibility across various species, including humans [93], monkeys [94,95], and rodents [96]. While the outcomes may vary depending on the specific compound and dosage used [97,98,99], these agonists have also been linked with neuroprotective effects, functional restoration, and notable improvement in working memory following TBI [100,101,102]. Conversely, studies involving the blockade of ADRA2A receptors by using Alpha-2A adrenergic antagonists have shown negative impacts on cognitive function. For instance, young adult monkeys exhibited impaired spatial working memory [103], and aged rats displayed deficits in delayed alternation performance [104]. These collective findings suggest a pivotal role for ADRA2A receptors in cognitive function. Of note, the LC sends afferent projections to the SN and even scattered afferents to the STR [105,106]; thus, it is possible that the observed alterations within these regions in this study may be a compensatory response to alterations in NA synthesis in the LC following msTBI. In line with this, striatal NA plays an important role in cognition, with LC–striatal NA connections key for response inhibition and cognitive flexibility [107]. However, the specific mechanisms via which TBI may subtly alter noradrenergic input to the nigrostriatal pathway from the LC and its downstream effects remain to be elucidated. In order to probe this further, future investigations should investigate changes in DβH within the LC, as well as concomitant changes in NA levels within the SN and STR, at chronic time points following experimental diffuse axonal injury. Unfortunately, due to the archival nature of the tissue, such investigations were outside the scope of the current study.

Accumulating evidence also suggests that LC–SN projections and NA play an important role in maintaining dopaminergic neurons in the SN [108,109,110,111]. Studies demonstrate that disturbance of the NA system is correlated with both the onset and progression of DA neuronal loss in PD [111,112,113,114]. This is, however, contrary to our findings, where alterations in the NA system were noted, with no concomitant changes in the DA system. These discrepancies may be attributed to a delayed response within the DA system. In line with this, a post-mortem study by Zarow et al. investigating 19 idiopathic PD cases suggests that neuronal loss in the LC-NA system is greater than in the SN-DA system, which corroborates Braak’s theory, where degeneration of LC-NA neurons occurs prior to SN-DA neurons [20,115]. Thus, it may be that the 12-month time point utilized in this study is insufficient to observe changes in the DA system, but rather earlier, more subtle changes in the NA system. This would also be consistent with the relatively mild cognitive changes observed in this cohort previously [73]. It is also important to acknowledge, however, that our analysis focused on a broad characterization of DA and NA changes using Western blot analysis of total protein levels for each marker of interest. It is possible that a more detailed analysis using RNAseq or microdialysis might reveal subtle changes following TBI that are not readily apparent at the gross protein level. Future studies should also investigate longer time points following TBI, such as 15 or 18 months post-injury, to investigate progressive alteration in the DA system, particularly within the SN and STR, in the face of the aging phenotype, and incorporate additional markers associated with these pathways, particularly the D2-like receptor family and the dopamine transporter (DAT).

Another notable finding of this study was the increase in DRD1 protein levels observed in the PFC of the mTBI group at 12 months post-injury compared to the sham and rmTBI groups. This increase in DRD1 expression might be associated with the previous findings in the elevated plus maze in this cohort of animals, with mTBI animals having reduced anxiety, as indicated by them spending more time in the open arms and exhibiting a significantly higher number of open arm entries and crossings when compared to msTBI animals [73,116]. A similar phenotype of reduced anxiety and hyperactivity has also previously been reported following mTBI across multiple other studies [117,118,119,120]. Elevated DRD1 expression could provide an explanation for this effect, as increased DRD1 activity has been linked to reduced anxiety [121]. Both overexpression of DRD1 [122] and optogenetic stimulation of DRD1, but not DRD2 [121], within the PFC, have shown similar reductions in anxiety-like behavior, as indicated by a higher number of open arm entries in the elevated plus maze [121,122]. DRD1 triggers a cascade of intracellular events through the adenylate cyclase/cyclic adenylate/protein kinase A (AC/cAMP/PKA) pathway [123,124], with the activity of PKA associated with anxiety [125]. In support of this, a study utilizing PKA inhibitor (H-89) demonstrated that injecting a high dosage of H-89 into medial PFC resulted in reduced anxiety symptoms [126]. Despite these findings, the reason why increased PFC DRD1 protein expression is present in the mTBI animals, and not in the other injury groups, remains unclear. It is possible that compensatory mechanisms are at play, where mTBI may induce recovery through DRD1 signaling. Nevertheless, direct investigation into DRD1 expression and its specific effects on anxiety and hyperactivity following chronic TBI is required for a more comprehensive understanding.

Based on the findings of this study regarding the changes in the DA and NA systems after TBI, it was pertinent to also explore the protein levels of the catecholamine-metabolizing enzyme, COMT. The activity of COMT is associated with the degradation of catecholamines, where lower activity can potentially lead to elevated neurotransmitter levels, while higher activity can potentially result in decreased neurotransmitter levels [127,128,129]. Indeed, COMT gene variants that alter dopamine levels are associated with cognitive decline in PD [130,131] and, similarly, impaired cognitive flexibility following TBI [127,132]. The investigation of COMT protein level following TBI is limited, with one study demonstrating that following focal TBI, both sCOMT and mbCOMT were increased in the ipsilateral cortex at 3 and 14 days post-injury [133], but longer time points have not been assessed to date. Here, at 12 months post-injury, regardless of the TBI severity, no changes were seen. However, it is important to emphasize that a more comprehensive understanding of the alterations in catecholamine metabolism following TBI might be gained through the additional examination of other catecholamine-metabolizing enzymes, such as monoamine oxidase and phenylethanolamine N-methyltransferase, or the incorporation of more sensitive methods, such as high-performance liquid chromatography (HPLC), to obtain a clearer insight into catecholamine levels and levels of their metabolites in response to TBI.

Interestingly, despite the subtle changes in catecholaminergic pathways discussed above, there was very little alteration in the glial response to injury in any of the brain regions examined, regardless of injury severity. In fact, the only significant finding relative to shams was a reduction in GFAP + cells in the SN of msTBI animals. Although it did not reach statistical significance relative to sham, there was also an increase in the total population of microglial cells in the STR following msTBI compared to rmTBI, with a significant main effect of injury noted. Interestingly, these microglia appeared to be in a resting/ramified state, rather than an activated/ameboid state. The underlying mechanism driving this trend towards a higher resting microglia number within the STR at 12 months post-msTBI remains unclear; yet, it is plausible that these cells are returning to their ramified state in the STR, indicating that this may indicate recovery from a previous state of increased neuroinflammation within this region. This notion could be supported by the concurrent increase in the levels of ADRA2A receptor in the STR, as ADRA2A is known to have anti-inflammatory effects, with administration of an agonist of this receptor shown to reduce the release of pro-inflammatory cytokines and downregulate pathways like NF-kB and NLRP3 inflammasome in the acute phase following TBI [101,134]. Additionally, a study investigating adrenergic receptor signaling in microglia suggests that the process of retraction is associated with ADRA2A, mainly in activated microglia rather than resting microglia [135]. However, it is important to note that several studies have indicated that the role of microglia cannot be solely characterized by a morphological change from a ramified to an amoeboid shape or vice versa [136,137,138], as was done in the current work. It is, thus, crucial that future studies more comprehensively probe the neuroinflammatory response, extending the analysis to include additional markers, such as CD16/32 and CD26, and further quantification of cytokine release via ELISA, in order to identify the phenotype of microglia and their release of pro-inflammatory cytokines, respectively. Further, as IBA1 does not distinguish between microglia and infiltrating macrophages, more specific markers, such as TMEM119 and P2RY12, either alone or in combination, may have utility for more nuanced probing of the microglial response. In support of this, a recent study of aged controls and individuals with AD found that the specific combination of these markers (i.e., phenotypes) differed significantly between these two groups [139].

In addition to the increased population of microglia in the STR following msTBI, there was a notable reduction in the population of GFAP+ astrocytes in the SN. Astrocytes can function in two primary states: resting and reactive. Under normal conditions, astrocytes play a crucial role in maintaining brain homeostasis by regulating the levels of reactive oxygen species, supporting neural development and survival, and providing structural support to mitochondria to maintain the energy level and integrity of neural circuits [140,141,142]. Studies have shown that deficiency in astrocytes can lead to the disruption of the blood–brain barrier seen in patients with PD. Further, in a rat model genetically modified for astrocyte ablation, about 50% more loss of cortical tissue was seen compared to wild-type rats following a moderate TBI, highlighting the neuron-protective role of astrocytes following injury [141]. Conversely, reactive astrocytes undergo morphological changes in response to injury or pathological conditions, which can exacerbate neuroinflammation and worsen secondary brain injury following TBI [143]. Interestingly, our analysis of astrocyte reactivity in the SN revealed that most of these cells were in a resting state, suggesting that they were not actively responding to injury or pathology and, instead, were likely providing support in maintaining the normal function of SN. Thus, in this context, the reduction observed in the total astrocyte population in the SN may be detrimental, even without a concomitant increase in astrocyte reactivity. In line with this, impairments in cognitive flexibility [73] and decreases in locomotion [80] have previously been noted in this cohort following msTBI.

In addition to the limitations associated with the analysis of archival tissue noted above, it is also important to acknowledge that the tissue utilized in this study was collected from animals who were approximately 15 months of age (i.e., 10–12 weeks at the time of injury; follow-up time point of 12 months post-injury). While difficult to exactly equate this to human year equivalents, based on their lifespan of ~2.5–3 years, Sengupta (2013) has offered calculations for rat–human age equivalents at each of their various “life phases” [75]. In general, across the lifespan, it is suggested that 13.2 rat days are equal to 1 human year. Thus, a 12-month-old rat would be equivalent to approximately a 30-year-old human, with an 18-month-old rat equivalent to approximately a 45-year-old human. The 15-month-old rats used within the current study would fall somewhere within this range and would, therefore, be not quite yet equivalent to true “middle age” in humans. Given that age is the biggest risk factor for PD [144], it may be that rats used in the current study were not yet old enough to observe post-injury changes in either catecholaminergic signaling or neuroinflammation that may set the stage for PD development. In line with this, the brain is known to switch to a pro-inflammatory state with increasing age, which may sensitize the brain to the effects of infection or insult (such as a TBI) (for review, see Sparkman and Johnson, 2008) [145]. Similarly, both SN/VTA-DA and LC-NA neuron density have been shown to progressively decrease with age [146], and post-mortem studies of NA extracted from homogenized brain tissue suggest that levels tend to be lower in older adults than in younger adults [147] (although, interestingly, plasma and CSF levels tend to be higher; for review, see Mather, 2022 [148]). Thus, future studies should investigate post-injury time points of 18 months or beyond in preclinical models or, alternatively, look at injury induced in older animals in order to investigate whether the aged phenotype may add a “second hit” to the system, exacerbating injury-related alterations in catecholaminergic signaling or neuroinflammation.

Similarly, in the current study, only tissue from male animals was available for analysis. This represents another potential limitation, as the markers of interest in the current study are known to vary with sex. For example, with regard to neuroinflammation, both the number and phenotype of microglia are known to differ between male and female rodents in a region- and age-dependent manner [149], with critical differences in function also noted (for review, see Han et al. 2021) [150]. Similar sex-related functional differences have also been noted for astrocytes (Santos-Galindo et al. 2011) [151]. Regarding catecholamine signaling, sex differences in LC structure and function have been widely reported to begin during puberty, with a larger LC volume, a higher number of NA neurons, and greater dendritic arbor density seen in female rodents. Sex differences have also been noted within midbrain dopaminergic regions, including both the SN and VTA, and have previously been elegantly reviewed by Glenda Gillies and colleagues [152,153,154]. Thus, it is plausible, and indeed likely, that there may be sex-related differences in how both catecholaminergic signaling and neuroinflammation are impacted by TBI and that this could result in different risk profiles for PD development following injury. In line with this, risk of developing PD is twice as high in males as in females [155] and there are significant differences in symptom presentation, risk factor profiles, and response to therapy in PD based on biological sex (for review, see Cerri et al., 2019 [156]). Thus, it is imperative that future work investigates these markers in females.

## 5. Conclusions

Taken together, this investigation represents the first comprehensive comparison of chronic alterations in catecholamine signaling and the glial response within the SN, STR, and PFC at 12 months following different severities of TBI. Although the changes were subtle at this time point, they nevertheless suggest a severity-dependent pattern. This underscores the nuanced and multifaceted nature of the brain’s response to different severities of diffuse axonal injury. Further, it emphasizes the need for continued exploration to unravel the time course and precise mechanisms via which TBI may impact catecholaminergic signaling following TBI, as well as the functional consequences of this. This is particularly critical given that, despite the subtlety of the noted changes, there were nevertheless alterations in NA signaling within the nigrostriatal pathway following msTBI, which could potentially set the stage for the later emergence of PD. While further experimental evidence is clearly required, this raises the intriguing possibility that monitoring such changes in survivors of TBI could provide an early indication of the risk of later PD development, allowing for earlier identification and potentially more effective therapeutic intervention.

## Figures and Tables

**Table 1:** Summary of neuroinflammatory marker results; changes following different TBI severities compared to shams at 12 months post-injury in the prefrontal cortex, striatum, and substantia nigra regions.

Marker Analysis	PFC		STR		SN	
	TBI Effect	Post hoc	TBI Effect	Post hoc	TBI Effect	Post hoc
Total GFAP+ cells	ns; *p* = 0.64	Δ^mTBI^ = −19.22 Δ^rmTBI^ = 12.85 Δ^msTBI^ = −6.70	ns; *p* = 0.3	Δ^mTBI^ = −44.45 Δ^rmTBI^ = −27.20 Δ^msTBI^ = −26.55	* *p* = 0.02	Δ^mTBI^ = −73.03 Δ^rmTBI^ = 52.48 Δ^msTBI^ = 121.9 *
% of Reactive Astrocyte	ns; *p* = 0.53	Δ^mTBI^ = −1.7 Δ^rmTBI^ = 0.71 Δ^msTBI^ = 0.13	ns; *p* = 0.08	Δ^mTBI^ = 0.25 Δ^rmTBI^ = 3.58 Δ^msTBI^ = 1.11	ns; *p* = 0.81	Δ^mTBI^ = −2.15 Δ^rmTBI^ = −0.75 Δ^msTBI^ = −1.18
Total IBA1+ cells	ns; *p* = 0.42	Δ^mTBI^ = 21.90 Δ^rmTBI^ = 4.67 Δ^msTBI^ = 6.38	* *p* = 0.02	Δ^mTBI^ = 8.32 Δ^rmTBI^ = 12.38 Δ^msTBI^ = −23.91 Δ^rmTBI-msTBI^ = −36.29 *	ns; *p* = 0.62	Δ^mTBI^ = 12.77 Δ^rmTBI^ = 24.57 Δ^msTBI^ = 9.06
% of Activated Microglial	* *p* = 0.02	Δ^mTBI^ = 3.04 Δ^rmTBI^ = 4.64 Δ^msTBI^ = −0.54 Δ^rmTBI-msTBI^ = −5.2 *	ns; *p* = 0.11	Δ^mTBI^ = 8.94 Δ^rmTBI^ = 7.70 Δ^msTBI^ = 4.43	ns; *p* = 0.11	Δ^mTBI^ = −4.98 Δ^rmTBI^ = −2.05 Δ^msTBI^ = −5.46

Note: Δ = mean of sham-mean TBI (s), negative value = increase value in mean 2 when compared to mean 1, positive value = decrease value in mean 2 when compared to mean 1, ns = not significant, * = *p* < 0.05. Bold text indicates statistical significance between treatment groups.

**Table 2:** Summary of catecholamine pathway marker results; changes following different TBI severities compared to shams at 12 months post-injury in the prefrontal cortex, striatum, and substantia nigra regions.

Marker Analysis	PFC		STR		SN	
	TBI Effect	Post hoc	TBI Effect	Post hoc	TBI Effect	Post hoc
TH	ns; *p* = 0.15	Δ^S-mTBI^ = 0.19 Δ^S-rmTBI^ = −0.19 Δ^S-msTBI^ = −0.32	ns; *p* = 0.38	Δ^S-mTBI^ = 0.23 Δ^S-rmTBI^ = 0.13 Δ^S-msTBI^ = 0.25	ns; *p* = 0.8	Δ^S-mTBI^ = −0.37 Δ^S-rmTBI^ = −0.14 Δ^S-msTBI^ = −0.13
DRD1	** *p* = 0.0013	Δ^S-mTBI^ = −0.86 ** Δ^S-rmTBI^ = −0.002 Δ^S-msTBI^ = −0.43 Δ^mTBI-rmTBI^ = 0.85 **	ns; *p* = 0.97	Δ^S-mTBI^ = 0.008 Δ^S-rmTBI^ = 0.03 Δ^S-msTBI^ = 0.06	ns; *p* = 0.94	Δ^S-mTBI^ = 0.015 Δ^S-rmTBI^ = −0.06 Δ^S-msTBI^ = 0.02
DβH	ns; *p* = 0.16	Δ^S-mTBI^ = −0.4 Δ^S-rmTBI^ = 0.26 Δ^S-msTBI^ = 0.29	ns; *p* = 0.29	Δ^S-mTBI^ = −0.27 Δ^S-rmTBI^ = −0.42 Δ^S-msTBI^ = −0.20	* *p* = 0.01	Δ^S-mTBI^ = 0.24 Δ^S-rmTBI^ = 0.14 Δ^S-msTBI^ = 0.89 ** Δ^rmTBI-msTBI^ = 0.74 *
ADRA1a	ns; *p* = 0.63	Δ^S-mTBI^ = −0.12 Δ^S-rmTBI^ = −0.11 Δ^S-msTBI^ = −0.05	ns; *p* = 0.86	Δ^S-mTBI^ = −0.03 Δ^S-rmTBI^ = 0.08 Δ^S-msTBI^ = 0.03	ns; *p* = 0.09	Δ^S-mTBI^ = 0.16 Δ^S-rmTBI^ = 0.20 Δ^S-msTBI^ = 0.12
ADRA2a	ns; *p* = 0.12	Δ^S-mTBI^ = −0.10 Δ^S-rmTBI^ = 0.21 Δ^S-msTBI^ = −0.44	* *p* = 0.02	Δ^S-mTBI^ = −0.006 Δ^S-rmTBI^ = −0.01 Δ^S-msTBI^ = −0.60 * Δ^rmTBI-msTBI^ = −0.59 *	ns; *p* = 0.68	Δ^S-mTBI^ = 0.33 Δ^S-rmTBI^ = 0.21 Δ^S-msTBI^ = 0.23
ADRB1	ns; *p* = 0.74	Δ^S-mTBI^ = 0.01 Δ^S-rmTBI^ = 0.01 Δ^S-msTBI^ = −0.04	ns; *p* = 0.09	Δ^S-mTBI^ = −0.1 Δ^S-rmTBI^ = 0.27 Δ^S-msTBI^ = 0.23	ns; *p* = 0.17	Δ^S-mTBI^ = 0.12 Δ^S-rmTBI^ = 0.03 Δ^S-msTBI^ = 0.06
mbCOMT	ns; *p* = 0.5	Δ^S-mTBI^ = 0.32 Δ^S-rmTBI^ = 0.30 Δ^S-msTBI^ = 0.80	ns; *p* = 0.72	Δ^S-mTBI^ = 0.27 Δ^S-rmTBI^ = −0.26 Δ^S-msTBI^ = 0.09	ns; *p* = 0.83	Δ^S-mTBI^ = −0.15 Δ^S-rmTBI^ = −0.09 Δ^S-msTBI^ = −0.13
sCOMT	ns; *p* = 0.92	Δ^S-mTBI^ = 0.12 Δ^S-rmTBI^ = 0.02 Δ^S-msTBI^ = 0.13	ns; *p* = 0.44	Δ^S-mTBI^ = −0.11 Δ^S-rmTBI^ = −0.34 Δ^S-msTBI^ = −0.18	ns; *p* = 0.35	Δ^S-mTBI^ = −0.25 Δ^S-rmTBI^ = −0.15 Δ^S-msTBI^ = −0.41

Note: Δ = mean 1 − mean 2, negative value = increase value in mean 2 when compared to mean 1, positive value = decrease value in mean 2 when compared to mean 1, S = Sham, mTBI = single mild TBI, rmTBI = repetitive mild TBI, msTBI = moderate–severe TBI, ns = not significant, * = *p* < 0.05, ** *p* < 0.01. Bold text indicates statistical significance between treatment groups.

**Table 3:** Regions of interest.

Region	Coronal Coordinates (Bregma) [81]		Region of Interest (Both Left and Right)
Prefrontal cortex	3.7 mm to 3.2 mm		Prelimbic Area Anterior cingulate area Infralimbic Area
Striatum	Early	1.0 mm to 0.48 mm	Caudoputamen
	Middle I	0.20 mm to −0.40 mm	
	Middle II	−0.80 mm to −1.30 mm	
	Late	−1.5 mm to −2.10 mm	
Substantia Nigra	Early	−4.5 mm to −5.2 mm	-Substantia nigra, compact part -Substantia nigra, reticular part
	Middle	−5.2 mm to −5.8 mm	
	Late	−5.8 mm to −6.3 mm	

**Table 4:** Antibodies investigated using immunohistochemistry.

Primary Antibody	Species	Conc.	Catalogue#	Analysis Target	Analysis Platform and Parameters
Ionized calcium-binding adaptor molecule 1 (IBA1)	Rabbit	1:20,000	Wako- 019-19741	Microglial reactivity	Halo microglial activation module: Min cell body diameter—3.4 μm ∙Contrast threshold—0.3 pixel ∙Min process OD—0.25 pixel ∙Max process Radius—12 μm ∙Max fragmentation length—2.5 μm ∙Activation process thickness—2.12 μm
Glial fibrillary acidic protein (GFAP)	Rabbit	1:40,000	Dako- Z0334	Astrocyte reactivity	Image J [version 1.53b]: ∙Manual identification of astrocyte morphology

**Table 5:** Primary antibodies investigated via Western blot.

Primary Antibody	Species	Conc.	Catalogue#	Analysis Target
Tyrosine Hydroxylase (TH)	Rabbit	1:1000	Abcam-ab112	Catalytic enzyme for conversion of tyrosine to DA
Dopamine Beta Hydroxylase (DβH)	Rabbit	1:500	Abcam-ab209487	Enzyme converts dopamine to norepinephrine
Dopamine receptor D1 (DrD1)	Rabbit	1:1000	Abcam-ab20066	Receptor from D1_R_ family
Rabbit anti-Dopamine receptor D4 (DrD4)	Rabbit	1:1000	Abcam-ab20424	Receptor from D2_R_ family
Rabbit anti-Catechol-O-methyltransferase (COMT)	Rabbit	1:1000	Abcam-ab226938	Enzyme that degrades catecholamines
Rabbit anti-alpha 1a Adrenergic receptor (ADRA1A)	Rabbit	1:1000	Abcam-ab137123	Alpha-1 adrenergic receptor subtypes
Rabbit anti-alpha 2a Adrenergic receptor (ADRA2A)	Rabbit	1:1000	Abcam-ab85570	Alpha-1 adrenergic receptor subtypes
Rabbit anti-beta 1 Adrenergic receptor (ADRB1)	Rabbit	1:1000	Abcam-ab3442	A beta-adrenergic receptor
Chicken anti-GAPDH	Chicken	1:10,000	Abcam-108162	Housekeeping protein

## Data Availability

The data that support the findings of this study can be made available upon request from the corresponding author.

## Supplementary Materials

The following supporting information can be downloaded at: https://www.mdpi.com/article/10.3390/molecules29071470/s1. Raw images of the Western blots for analysis presented in Figures S1–S8.
