# Supplementary material for: Long-Term Impact of Diffuse Traumatic Brain Injury on Neuroinflammation and Catecholaminergic Signaling: Potential Relevance for Parkinson’s Disease Risk
Source: Molecules. 2024 Mar 26;29(7):1470. doi: 10.3390/molecules29071470 (PMC11013319; doi:10.3390/molecules29071470)

Supplementary Figure S1: Microglial analysis based on morphology parameters in the HALO platform

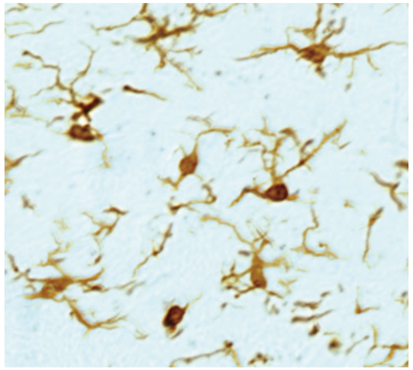

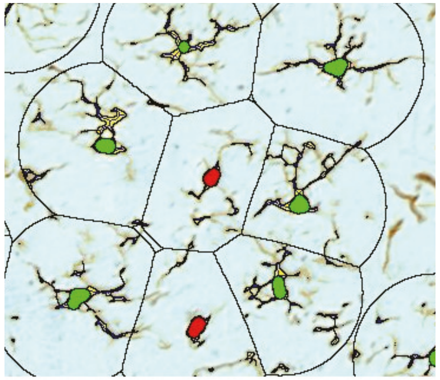

Representative image of microglial analysis: (left) Original image; (right) microglial analysis using the Halo platform and parameters outlined in Table 2. In the analysis, red represents activated microglia, green represents resting microglia, and yellow indicates processes. It is crucial to note that accuracy is targeted at 75%, considering staining intensity, tissue quality, and inevitable factors.

The same control was applied across different blots to serve as a standard. C: Control; S: Sham; M: single mild TBI; RM: Repetitive mild TBI; MS: Moderate-Severe TBI.

Supplementary Figure S2: Tyrosine Hydroxylase (TH) Western Blot Analysis

Prefrontal Cortex

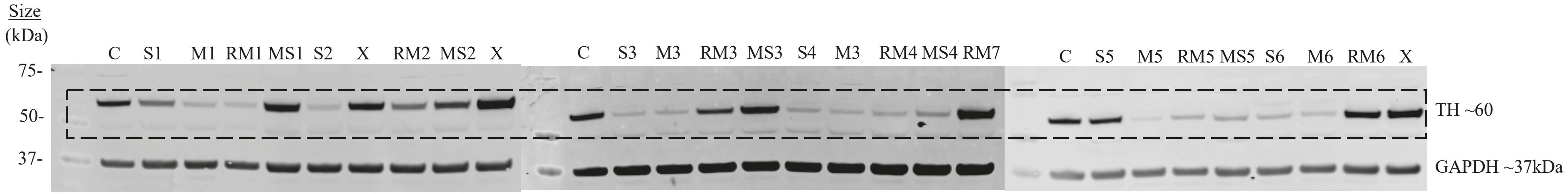

Striatum

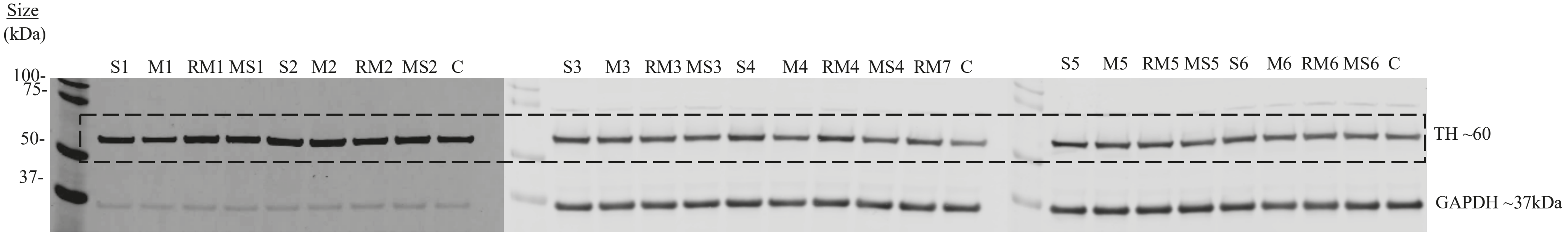

Substantia Nigra

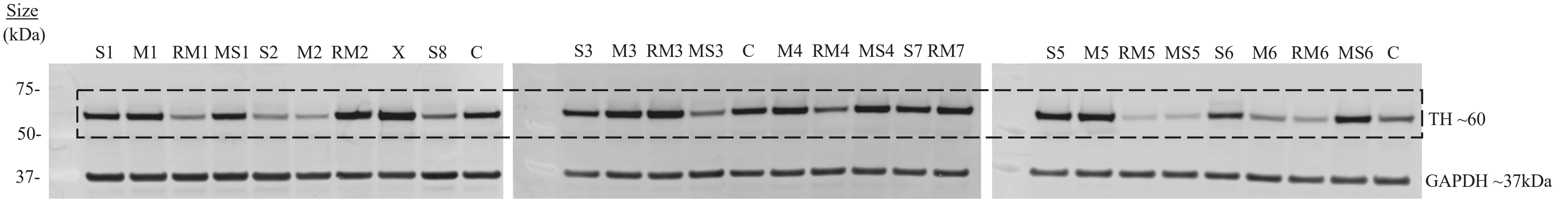

Supplementary Figure S3: Dopamine Receptor D1 (DRD1) Western Blot Analysis

Prefrontal Cortex

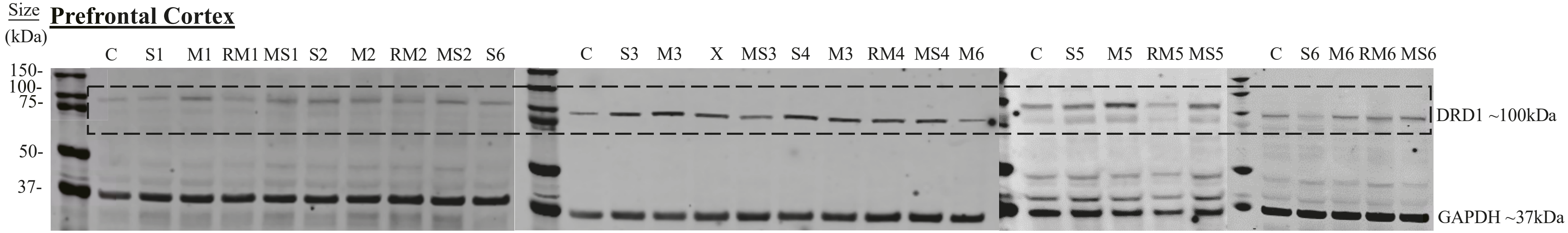

Striatum

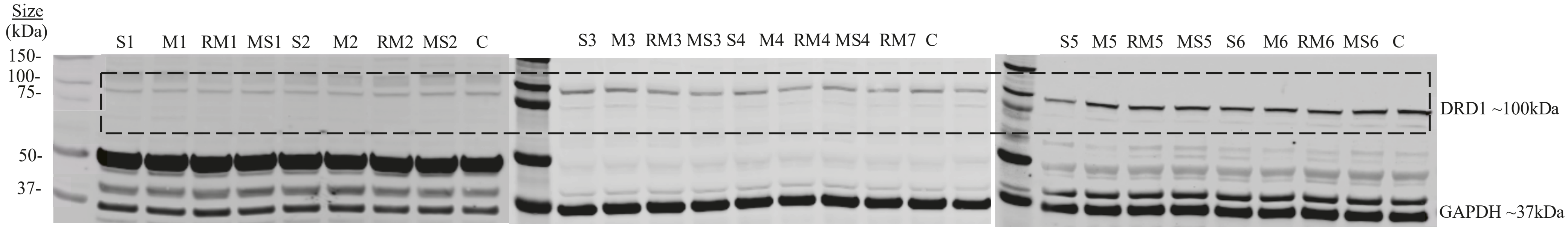

Substantia Nigra

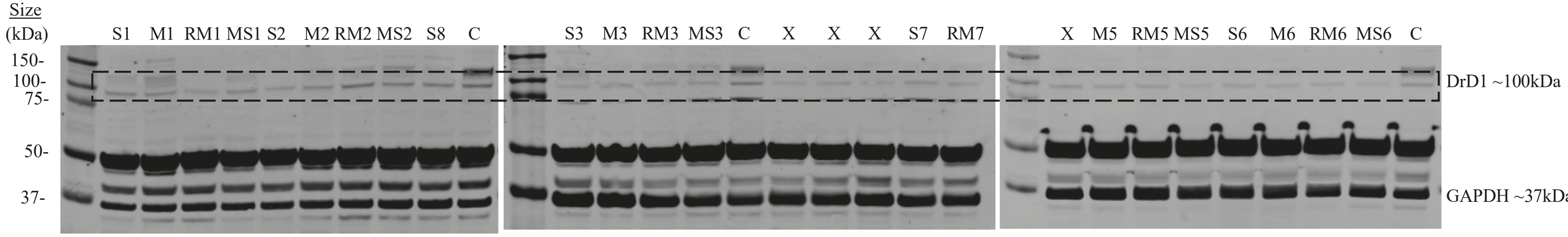

Supplementary Figure S4: Dopamine Beta Hydroxylase (DβH) Western Blot Analysis

Prefrontal Cortex

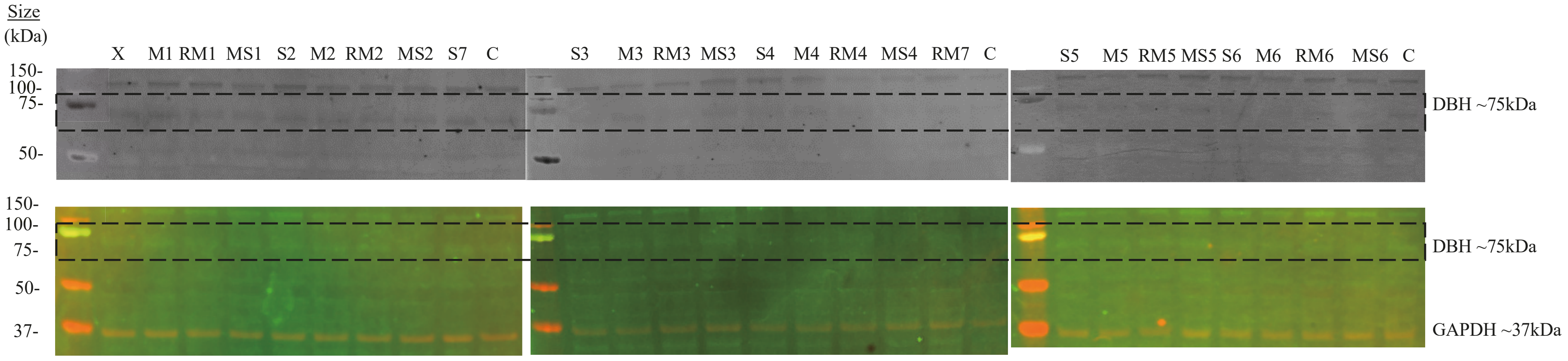

Striatum

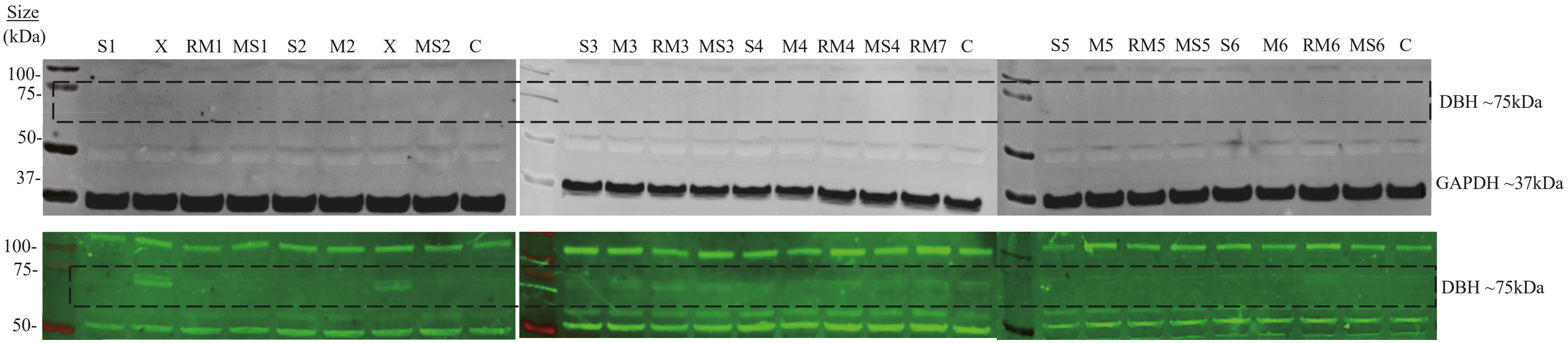

Substantia Nigra

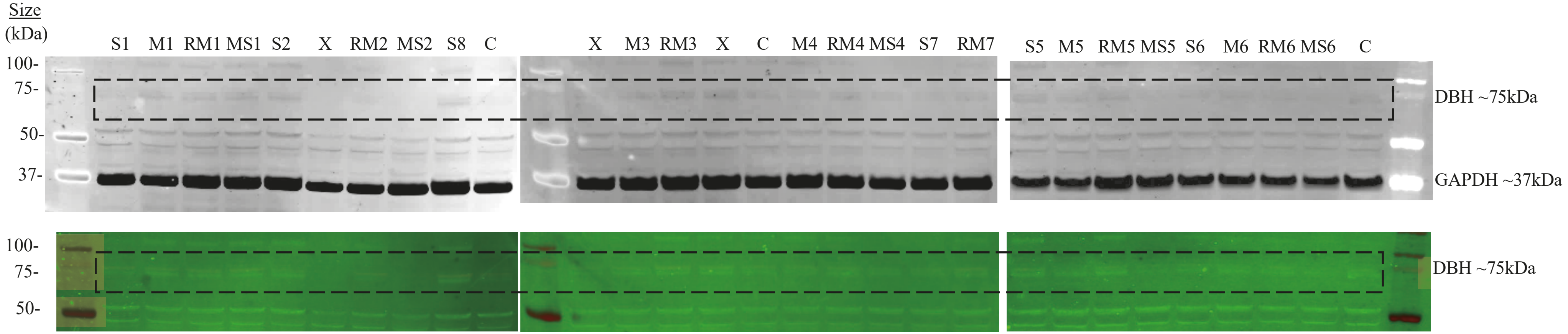

Supplementary Figure S5: Adrenoreceptor Alpha 1a (ADRA1a) Western Blot Analysis

Prefrontal Cortex

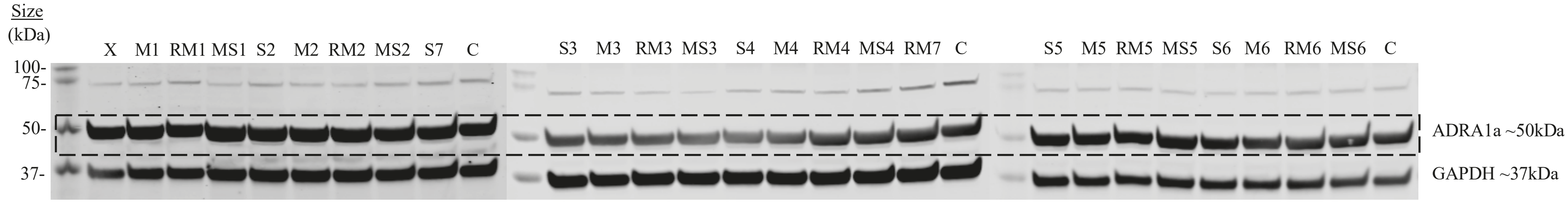

Striatum

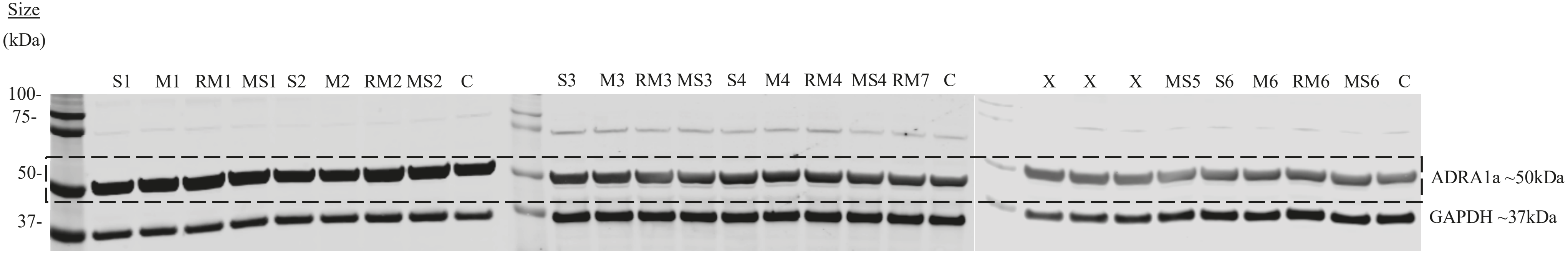

Substantia Nigra

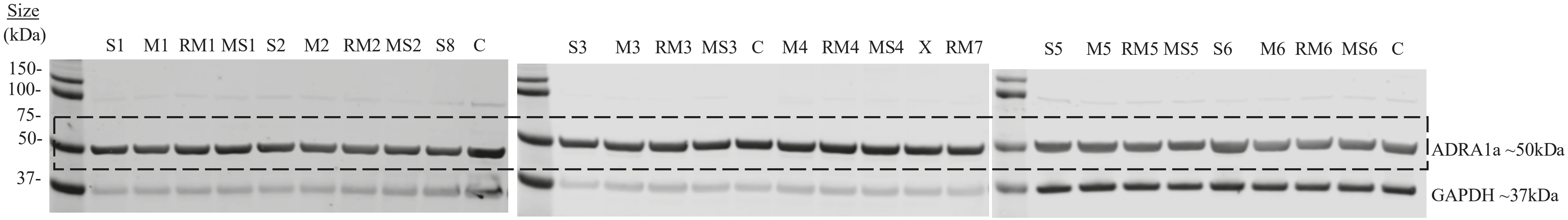

Supplementary Figure S6: Adrenoreceptor Alpha 2a (ADRA2a) Western Blot Analysis

Prefrontal Cortex

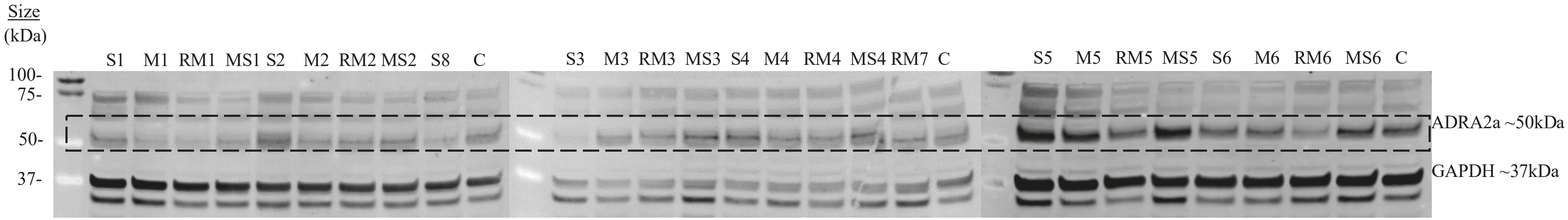

Striatum

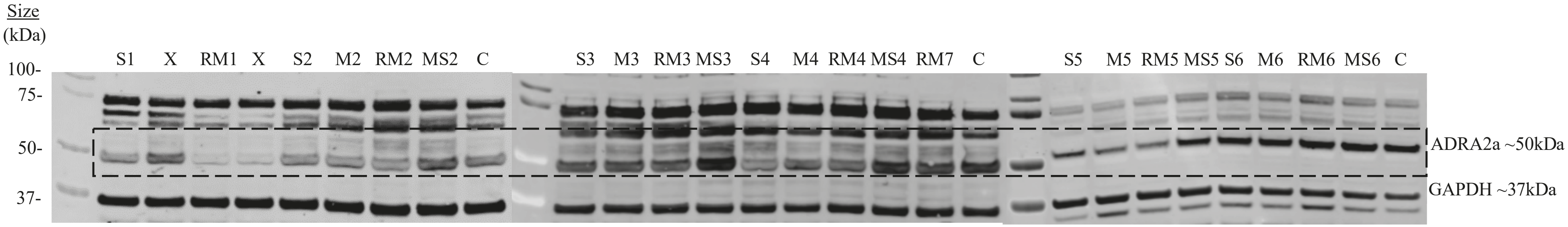

Substantia Nigra

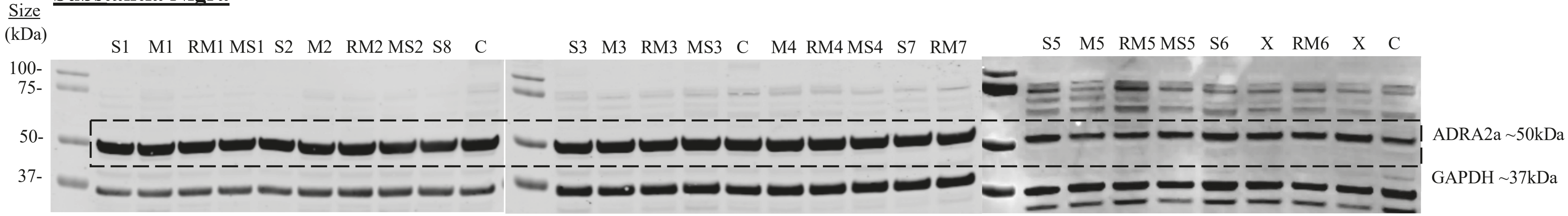

Supplementary Figure S7: Adrenoreceptor Beta 1(ADRB1) Western Blot Analysis

Prefrontal Cortex

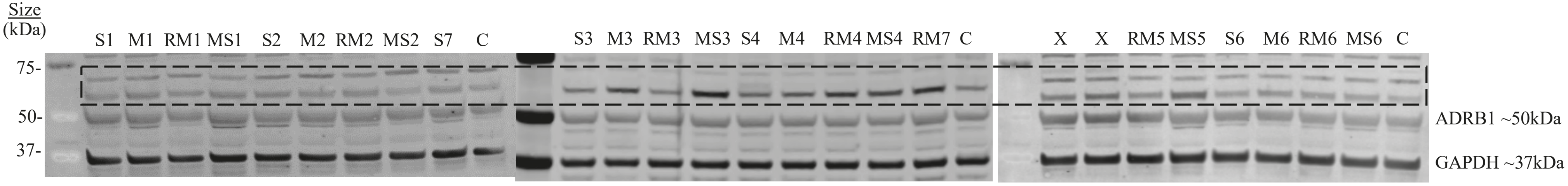

Striatum

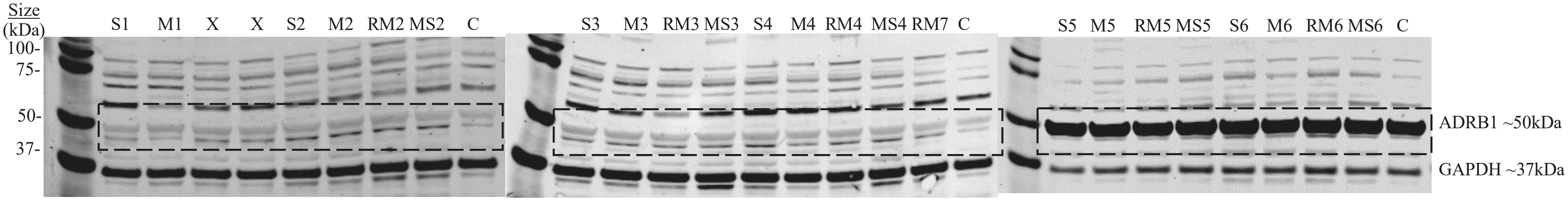

Substantia Nigra

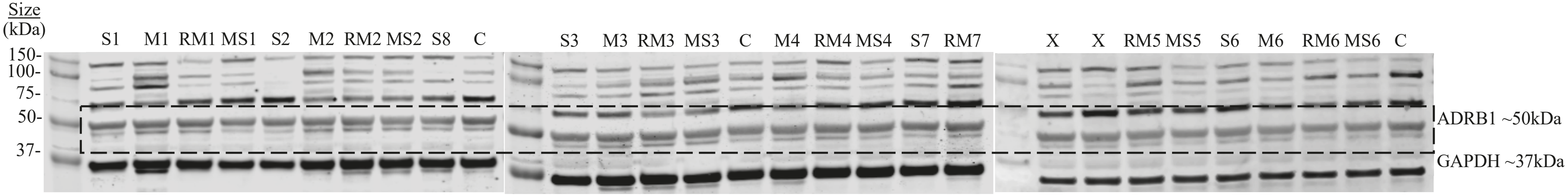

Supplementary Figure S8: Catechol-O-methyltransferase (COMT) Western Blot Analysis

Prefrontal Cortex

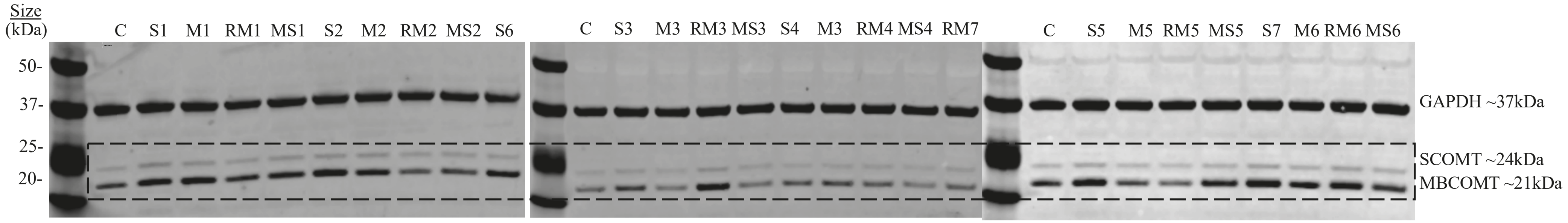

Striatum

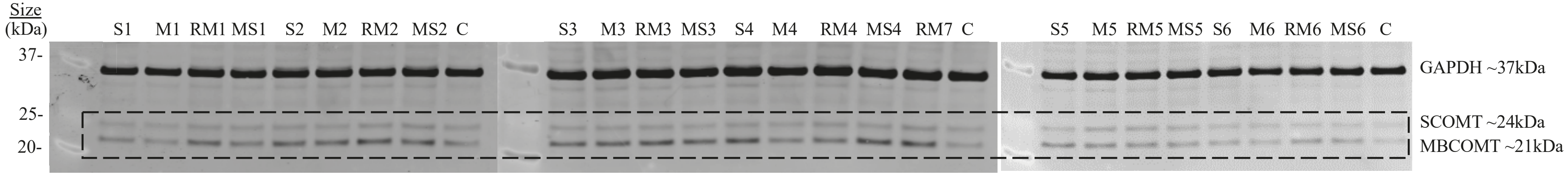

*M1 excluded for mbCOMT analysis

Substantia Nigra

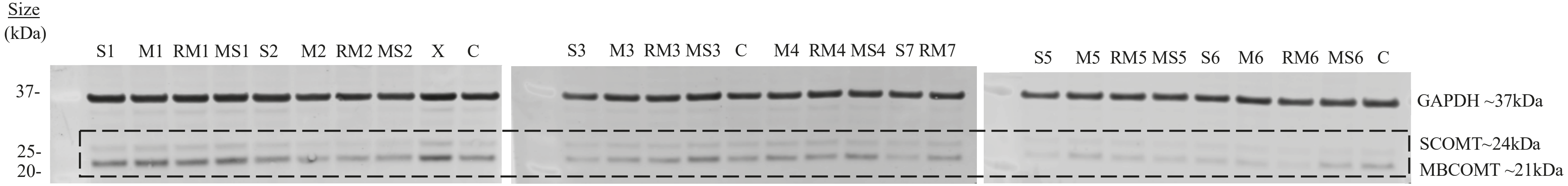

Supplement: Supplementary file 1 [file molecules-29-01470-s001.zip › molecules-2835043-supplementary.pdf]
